# Postoperative Albumin Drop Is a Marker for Surgical Stress and a Predictor for Clinical Outcome: A Pilot Study

**DOI:** 10.1155/2016/8743187

**Published:** 2016-01-06

**Authors:** Martin Hübner, Styliani Mantziari, Nicolas Demartines, François Pralong, Pauline Coti-Bertrand, Markus Schäfer

**Affiliations:** Department of Visceral Surgery and Transplantation and Department of Endocrinology, Diabetology and Metabolism, Lausanne University Hospital (CHUV), Rue du Bugnon 46, 1011 Lausanne, Switzerland

## Abstract

*Background*. Surgical stress during major surgery may be related to adverse clinical outcomes and early quantification of stress response would be useful to allow prompt interventions. The aim of this study was to evaluate the acute phase protein albumin in the context of the postoperative stress response.* Methods*. This prospective pilot study included 70 patients undergoing frequent abdominal procedures of different magnitude. Albumin (Alb) and C-reactive protein (CRP) levels were measured once daily starting the day before surgery until postoperative day (POD) 5. Maximal Alb decrease (Alb Δ min) was correlated with clinical parameters of surgical stress, postoperative complications, and length of stay.* Results*. Albumin values dropped immediately after surgery by about 10 g/L (42.2 ± 4.5 g/L preoperatively* versus *33.8 ± 5.3 g/L at day 1, *P* < 0.001). Alb Δ min was correlated with operation length (Pearson *ρ* = 0.470, *P* < 0.001), estimated blood loss (*ρ* = 0.605, *P* < 0.001), and maximal CRP values (*ρ* = 0.391, *P* = 0.002). Alb Δ min levels were significantly higher in patients having complications (10.0 ± 5.4* versus *6.1 ± 5.2, *P* = 0.005) and a longer hospital stay (*ρ* = 0.285, *P* < 0.020).* Conclusion*. Early postoperative albumin drop appeared to reflect the magnitude of surgical trauma and was correlated with adverse clinical outcomes. Its promising role as early marker for stress response deserves further prospective evaluation.

## 1. Introduction

Surgical interventions trigger metabolic stress responses of different magnitude, which contribute to complication rate, delayed recovery, and length of hospital stay. Primarily, the term “major abdominal surgery” remains poorly defined and includes procedure-related factors, like the type of surgical approach (laparoscopy* versus* laparotomy), type and extent of organ resection, operative time, and blood loss; but secondarily also patient-related factors, for example, underlying disease (benign* versus* malignant), nutritional status, and preexisting comorbidities, are subsumed [1–7].

Recent improvements in perioperative care aiming to modulate an overwhelming stress response have been proven to be effective. Application of enhanced recovery pathways (ERAS) has shown a decreasing effect on surgical stress and subsequently reducing complications, hospital stay, and costs after colorectal surgery [8–10]. A strict perioperative nutritional support and the use of immune-modulating formulas proved to reduce both (infectious) complications after major surgery and hospital stay. Similarly, the perioperative use of corticosteroids has been advocated recently; and preliminary results for this simple intervention are promising with regard to postoperative outcomes [11–14].

A simple and reliable parameter representing surgical stress would be clinically important to identify patients at risk and to tailor perioperative care. Thorell et al. proposed insulin resistance to quantify the stress response [15, 16]. However, insulin resistance is difficult to measure and costly and has hence never been implemented in clinical routine. For the same reasons, interleukin-6, interleukin-10, and other cytokines are used in clinical studies only [2, 4, 6, 7, 17]. Postoperative serum C-reactive protein (CRP) levels are widely used in clinical practice to assess postoperative inflammation and to predict postoperative complications [18]. A major drawback of CRP as predictor for stress-related complications is its slow kinetics [2, 7, 17, 19]. Peak values are measured only at postoperative day 2 or 3, which may be too late for early preventive interventions. Albumin is the most abundant protein in humans and widely used as nutritional marker and predictor for outcomes. In addition, albumin shows an immediate response to surgical stress and could therefore qualify to measure surgical stress and to predict a complicated postoperative course [20–22]. This particular aspect has not yet been considered for clinical use.

The aim of the present study was therefore to assess serum albumin levels as response marker for surgical stress response and potential predictor of adverse outcomes.

## 2. Materials and Methods

A prospective observational pilot study was conducted in the Department of Visceral Surgery, University Hospital of Lausanne (CHUV), in order to compare the perioperative metabolic profiles of patients undergoing abdominal surgery. The present analysis focuses on the acute response of albumin in the postoperative phase and its predictive potential for clinical outcome. The study was approved by the local ethical committee (Protocol number 273/11) and all included patients signed the consent form.

The study cohort included 70 patients recruited from December 2011 to July 2012. These were ten consecutive patients for seven current and representative abdominal procedures of different magnitude: laparoscopic cholecystectomy, extraperitoneal incisional hernia repair, laparoscopic and open colectomy, upper GI resections (gastrectomy or esophagectomy), and liver and pancreas resections.

Serum albumin (g/L) and C-reactive protein (CRP, mg/dL) levels were measured in a fasting state at 7 a.m. starting the day before surgery until postoperative day (POD) 5 in a standardized manner according to hospital technical guidelines. Samples on POD 0 were taken 4–6 hours postoperatively. Pertinent demographics, surgical parameters, and clinical outcome measures were prospectively recorded in anonymized form in a computerized database. Operation (OR) duration was measured by the anesthetist from incision to skin closure. Intraoperative blood loss was estimated (EBL) as joint decision (anesthetist, surgeon) by measuring aspiration fluids and soaked gauze materials. Postoperative complications were graded by severity by use of the validated Dindo-Clavien system; grades I-II were considered as minor and III-IV were considered as major complications, respectively. Mortality was documented as grade V [23]. Hospital stay was counted from the day of surgery.

Normal and nonnormal continuous variables were compared using Student's *t*-test and Mann-Whitney *U* test, respectively. ANOVA test was employed for comparing multiple groups. Statistical correlation of means was tested using Pearson's test. A *P* value < 0.05 was considered to be statistically significant. Data analysis was performed with the Statistical Package for the Social Sciences (SPSS 14.0, Inc., Chicago, IL).

## 3. Results

Demographic and surgical details of the study cohort are displayed by type of surgery in Table 1. Postoperative complications are shown in detail for each group in Table 2, according to the Dindo-Clavien classification [23].

### 3.1. Metabolic Stress Response (Figure 1)

CRP levels were 6.0 ± 9.4 mg/dL preoperatively. They increased slowly after surgery and peaked at POD 2 (149.6 ± 88 mg/dL, *P* < 0.001) before slowly decreasing again. Albumin values dropped immediately after surgery by about 10 g/L (42.2 ± 4.5* versus *33.8 ± 5.3 g/L, *P* < 0.001) and showed a very slow recovery. Hematocrit levels decreased in the same time period from 38.6 ± 10.8% to 35.3 ± 9.0% (*P* < 0.001); the resulting difference of 3.1 ± 4.4% corresponded to a relative postoperative decrease of 7.1%.

### 3.2. CRP versus Albumin: Correlation and Differences

**Figure 1:**
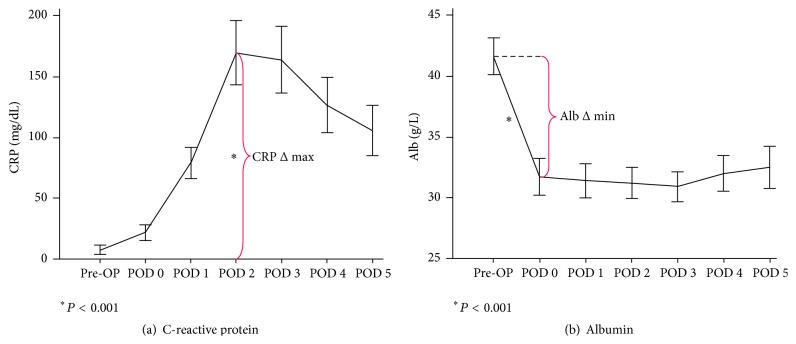
Acute inflammatory response after abdominal surgery as reflected by albumin and CRP kinetics. C-reactive protein (CRP) and albumin were measured preoperatively (pre-OP), 6 hours after surgery, and on postoperative days (POD) 1–5. CRP levels peaked (CRP Δ max) on POD 2 (a), while minimal albumin levels (Alb Δ min) were measured already 6 hours after surgery (b).

**Figure 2:**
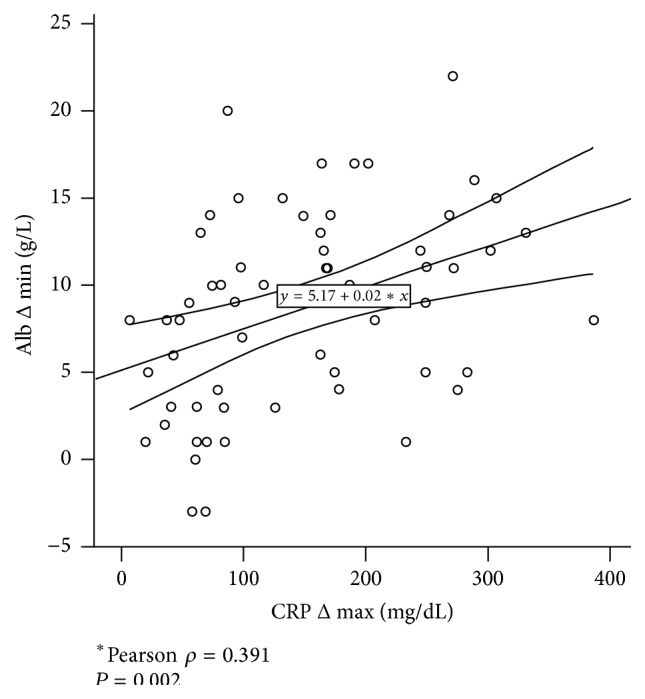
Correlation of postoperative CRP increase and albumin drop. Maximal C-reactive protein levels (CRP Δ max) are plotted against postoperative albumin drops (Alb Δ min).

**Figure 3:**
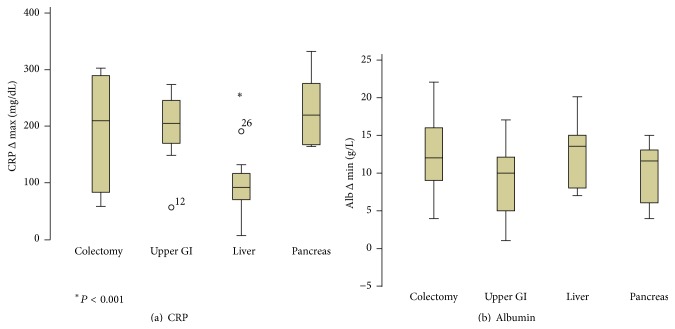
CRP levels and Albumin drop after different major surgery procedures. Maximal C-reactive protein levels (CRP Δ max) for major colon, upper gastrointestinal, liver, and pancreatic resections (a) showing significant lower values for liver resections. Postoperative albumin decrease (Alb Δ min) was equally high for the four different types of surgery (b).

Maximal CRP increase (CRP Δ max) and albumin decrease (Alb Δ min) were significantly correlated with each other (Pearson *ρ* = 0.391, *P* = 0.002) as shown in Figure 2. Striking differences were noted though between the two parameters. (I) Baseline CRP levels were close to 0 for most of the patients, while baseline albumin showed greater variation. (II) Maximal amplitude was reached for albumin 4–6 h after surgery and for CRP on POD 2 only (Figure 1). (III) The magnitude of four representative open major resections appeared to be equally represented by albumin measurements while the surgical stress of liver resections was underestimated by CRP levels (liver* versus* other: 98.0 ± 13.6* versus *202.8 ± 13.3 mg/dL, *P* < 0.001; Figures 3(a) and 3(b)).

### 3.3. Correlation of Albumin Drop with Surrogate Parameters for Surgical Magnitude

**Figure 4:**
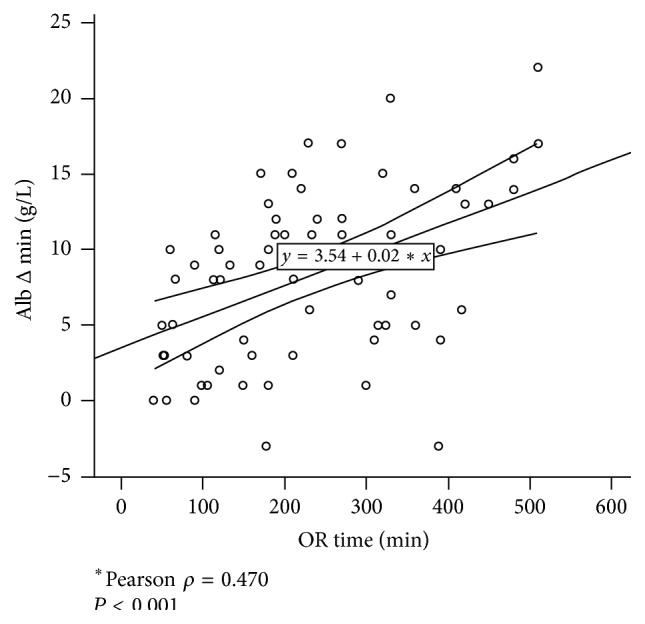
Correlation between albumin drop and operative time. Postoperative albumin decrease (Alb Δ min) is significantly correlated with operative time (Pearson *ρ* = 0.470, *P* < 0.001).

Median OR duration and estimated blood loss were 200 min (IQR 217) and 150 mL (IQR 400), respectively, for the entire cohort. Albumin decrease showed linear correlation to OR duration (Figure 4, Pearson *ρ* = 0.470, *P* < 0.001) and estimated blood loss (online appendix available online at http://dx.doi.org/10.1155/2016/8743187, Pearson *ρ* = 0.605, *P* < 0.001).

### 3.4. Correlation of Albumin Drop with Clinical Outcome Measures

**Figure 5:**
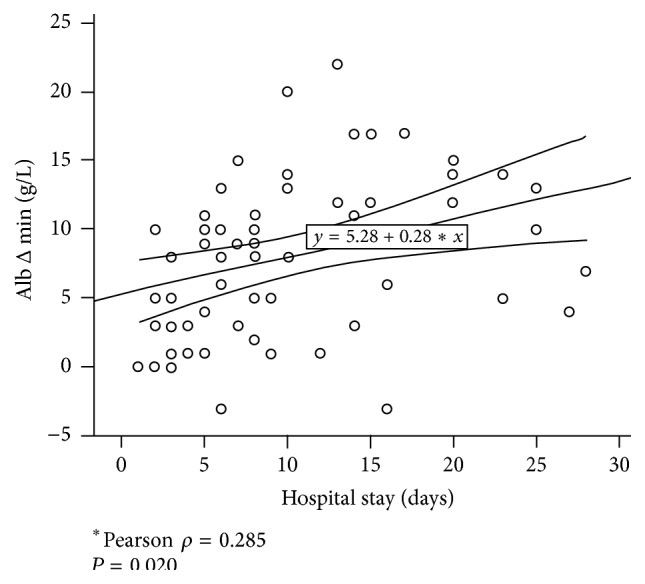
Albumin drop and hospital stay. Alb Δ min shows a positive correlation with increased length of hospital stay (Pearson *ρ* = 0.285,  *P* = 0.02).

Alb Δ min values were estimated for all patient groups in relation to postoperative complications (online appendix). There was a trend of higher Alb Δ min levels in patients with major complications (*P* = 0.082) and a significant difference comparing patients with no complication with the group with any complication (6.1 ± 5.2* versus *10.0 ± 5.4 g/L, *P* = 0.005). Albumin drop and length of hospital stay were statistically correlated (Pearson *ρ* = 0.285, *P* < 0.020) as shown in Figure 5.

## 4. Discussion

This pilot study evaluated serum albumin kinetics as response marker for surgical stress and predictor of adverse postoperative outcome of seven different surgical interventions. Our findings suggest that postoperative albumin decrease (Alb Δ min) reliably quantifies the magnitude of surgery and may be used as predictor for a complicated postoperative course. It can be assessed as early as 4–6 hours after surgery.

Major surgery is followed by an important metabolic stress response, which is closely related to adverse outcomes [2, 3, 7, 15]. A number of perioperative interventions allow modulating an excessive stress response, some of them having an important positive impact on clinical outcome [1, 5, 7, 9, 11, 24, 25]. Therefore, reliable prediction of surgical stress response is of high interest. The* ideal marker* has to be* easy* to measure, available* early* in the perioperative course, and* inexpensive*. It should be strongly* correlated with the extent of surgical trauma* and be a* reliable predictor* of complications and prolonged hospital stay. So far, no such parameter is available [4, 7, 26].

Stress response after surgery and trauma has been extensively studied and it involves important hormonal, electrolytic, and metabolic changes and liberation of cytokines [2, 6, 27, 28]. IL-6 is a proinflammatory cytokine that correlates with postoperative insulin resistance [29–31] and the magnitude of (surgical) trauma and increases hours after the hit [6, 27, 32, 33]. However, sophisticated and expensive measuring renders its routine use impracticable.

The role of acute phase proteins was investigated in our study by means of CRP levels and the decrease of serum albumin. CPR levels correlate closely with the magnitude of surgery and are routinely assessed to monitor postoperative systemic inflammatory response [1–3, 7, 17, 19]. The use of CRP levels on POD 4 has also been advocated as predictor for (infectious) complications [18]. However, kinetics are rather slow, and plasma peaks are only reached between POD 2 and POD 3. This is an important limitation, as potential therapeutic interventions should be launched as early as possible. Furthermore, CRP is produced in the liver, and its production is significantly impaired in patients with liver disease or after hepatectomy as shown in this present study. CRP may therefore not be an appropriate marker for surgical stress in patients undergoing liver surgery [19].

*Serum albumin* is widely used as reliable indicator for nutritional status and as predictor for clinical outcomes. The present study focused on the potential value of* early postoperative serum albumin drop* to measure surgical magnitude and predict the related adverse outcomes. Albumin accounts for 55–60% of protein in human plasma. About two-thirds of the total body albumin pool is in the extravascular space; exchange with the intravascular compartment (30–40%) takes place under physiological conditions at a rate of about 5%/h. Albumin is produced exclusively in the liver representing 50% of the organ's protein synthesis and can be upregulated up to threefold if the oncotic pressure decreases. Albumin degradation takes place in various organs, in a rate of about 5% per day given a half-life of 19 days; of note, plasma half-life of injected albumin is only one day due to redistribution into the extravascular compartment. Thus, serum albumin concentration depends basically on three factors, synthesis, distribution, and degradation [20, 34].

The combination of serum CRP and albumin changes has been correlated with adverse clinical outcomes. For instance, the Glasgow Prognostic Score (GPS) and modified GPS have been described for the prediction of survival in surgical patients, assigning to elevated CRP and lowered albumin levels a clearly negative prognostic value [35, 36]. Recently, Ishizuka and colleagues [37] found a significant correlation of an elevated CRP to albumin ratio with negative oncological outcomes in colorectal cancer patients, and Ranzani et al. [38] confirmed similar results in septic patients. The CRP/albumin ratio combines indeed information on nutritional and inflammatory status and could thus be a reliable marker of negative outcome. Our study confirms the aforementioned hypothesis and also focuses on the promptness of albumin decrease only hours after the surgical trauma, which has not yet been assessed and validated in the clinical setting.

Protein metabolism is significantly disturbed after any kind of traumatic event, for example, surgery, sepsis, and burn injuries [39, 40]; albumin has been identified as a reliable indicator of this process [34, 40, 41]. Plasma concentrations of albumin reveal an important decrease as early as a few hours after the hit [21, 34, 40, 41]. Yet, conflicting results from experimental and human studies still hamper a complete understanding of underlying pathophysiological mechanisms. (I) The importance of acute phase proteins during the early postoperative phase impairs* hepatic albumin synthesis,* to facilitate the production of these acute phase molecules (CRP, fibrinogen, and macroglobulin) needed in the host defense process [2, 40]. However, this initial decrease is transitory. Fractional albumin synthesis increases in the early postoperative period proportionally to the degree of inflammation; production can be further stimulated by perioperative nutrition [34]. (II) Basal energy expenditure increases nearly 10-fold in the early postoperative phase, and up to 20% of the body's store in protein can be* consumed* within ten days to favor glyconeogenesis [27]. (III) The most important postoperative losses of albumin however are due to* sequestration into the third space*. Capillary leak is a well-known phenomenon in the context of sepsis and (surgical) trauma [3, 28, 42]. Fleck et al. described already in 1985 that transcapillary exchange rate increased by 100% after major surgery and up to 300% in patients with septic shock [20, 41]. The increase in capillary escape rate occurs only hours after the offending trauma and can be twice as high in cachectic patients [20–22]. Perioperative nutrition will therefore decrease albumin losses not only by increasing hepatic synthesis as described above but also by decreasing losses in the extravascular space [20, 34]. Since the physiological efflux rate of 5%/h already represents about 10 times the rate of albumin synthesis and catabolism, it appears that capillary leak due to the metabolic stress response probably represents the principal mechanism of postoperative albumin drops [28, 34, 41]. A previous study estimated that 77% of the postoperative albumin decrease was due to redistribution, while 18% and 6% were attributed to blood loss and catabolism, respectively, [22]. Of note, transcapillary exchange rate clearly depended on the underlying trauma, and no increase was noted after minor surgery [20, 22].

Hemodilution is a potential confounder that needs to be taken into the equation of postoperative albumin decrease. In the present study, albumin dropped immediately by 20% from the baseline values, while hematocrit values diminished in the same time period by 7% only. This suggests that hemodilution played only a subsidiary role in postoperative albumin decrease, an observation consistent with previously published data [21, 22]. Also, the recent* ALBIOS* trial failed to demonstrate any benefit of albumin substitution in septic patients [43], suggesting that postoperative hypoalbuminemia represents an indicator rather than a cause of adverse outcomes.

In our study, albumin was strongly correlated with CRP levels but was available earlier in the postoperative course. It was strongly correlated with reliable surrogate parameters of the extent of surgery (OR duration, blood loss). The slow recovery of serum albumin during the first five postoperative days confirms previously published findings of Rittler et al. [40], who described a normalization of serum albumin levels by progressive reduction of initially increased albumin synthesis after open rectal resection.

Further, high Alb Δ min levels were associated with postoperative morbidity and prolonged hospital stay. Ryan et al. recently studied albumin changes one day after esophagectomy and associated low serum albumin <20 g/L with increased morbidity, longer intensive care unit stay, and higher reoperation rates [21]. Of note, the absolute baseline value of albumin can be influenced by preoperative hypoalbuminemia and thus malnutrition, which is an established risk factor for adverse outcomes [44]. Therefore, the postoperative decrease in albumin in relation to its preoperative levels appears to be more reliable. Furthermore, albumin drop can already be measured hours after surgery, as in our study, and not only on the day after [20–22, 41]. This is of importance as several interventions exist to attenuate the postoperative metabolic stress response [4, 7, 11, 14, 24, 25] and thus early identification of patients at risk is appealing. Lastly in practical terms, serum albumin is part of the routine work-up before major surgery and inexpensive and seems to reflect metabolic stress even after liver surgery to the same extent as after other major organ resections (Figure 3(b)).

Several limitations need to be addressed. This pilot study aimed at studying metabolic profiles after different surgical procedures. The modest and heterogeneous study cohort did not allow identifying meaningful cut-off values for albumin drop by means of a receiver operating characteristic (ROC) curve and hence calculating sensitivity, specificity, and predictive values. Our findings concerning albumin's role as acute phase protein need to be tested in larger patient samples to identify meaningful cut-off values and to guide therapeutic measures. These are the aims of our ongoing large prospective cohort study (NCT02356484). It remains further to be determined whether postoperative albumin decrease could be useful also for other types of surgeries or critically ill patients.

## 5. Conclusion

In summary, postoperative decrease in serum albumin was found in the present study to reflect the magnitude of surgery and the associated stress response. Whilst hypoalbuminemia (*absolute serum values*) is well established for nutritional assessment and prediction of complications, the early postoperative albumin decrease (*delta from baseline*) has not yet been implemented into clinical management. Postoperative albumin drops were strongly correlated with CRP increase but could already be measured 4–6 hours after surgery; albumin response was further related to clinical outcomes. This observation could be of clinical importance, since modulation of an overshooting stress response can be achieved by pharmacological and nutritional interventions as well as by specific modifications in perioperative care. This hypothesis needs confirmation and is currently tested in a prospective cohort study (NCT02356484).

## Supplementary Material

As illustrated in these two figures, postoperative albumine drop was significantly correlated with intraoperative blood loss (a), as well as the appearance of postoperative complications (b).

## Figures and Tables

**Table 1:** Demographics and surgical details.

	Cholecystectomy	Incisional hernia	Laparosc. colon res.	Open colon res.	Upper GI resection	Liver resection	Pancreas resection
Age (years)	61 ± 14	74 ± 10	57 ± 14	67 ± 9	49 ± 13	61 ± 16	64 ± 20
Gender (m/f)	6/4	6/4	7/3	7/3	8/2	7/3	7/3
BMI (kg/m^2^)	28 ± 3	27 ± 6	26 ± 7	27 ± 4	25 ± 4	24 ± 3	25 ± 3
OR time (min)	65 ± 21	121 ± 48	201 ± 66	305 ± 120	293 ± 112	283 ± 111	329 ± 100
EBL (mL)	0	28 ± 67	79 ± 80	410 ± 448	312 ± 203	1039 ± 1463	550 ± 428

Values are expressed as mean ± SD.

BMI: body mass index; OR time: operation room time; EBL: estimated.

**Table 2:** Complications according to Dindo-Clavien, according to the type of surgery [23].

	No complication	Minor complication (I-II)	Major complication (III-IV)	Mortality (V)
Cholecystectomy	10	0	0	0
Incisional hernia	4	5	1	0
Laparoscopic colectomy	6	4	0	0
Open colectomy	2	4	4	0
Upper GI resections	3	5	2	0
Liver resections	3	3	4	0
Pancreatic resections	1	8	0	1

## Abbreviations

Alb: Albumin
CRP: C-reactive protein
POD: Postoperative day
Alb Δ min: Maximal albumin decrease.
