# Supplementary material for: Postoperative Albumin Drop Is a Marker for Surgical Stress and a Predictor for Clinical Outcome: A Pilot Study
Source: Gastroenterol Res Pract. 2016 Jan 6;2016:8743187. doi: 10.1155/2016/8743187 (PMC4736779; doi:10.1155/2016/8743187)
Supplement: Supplementary file 1 — As illustrated in these two figures, postoperative albumine drop was significantly correlated with intraoperative blood loss (a), as well as the appearance of postoperative complications (b). [file 8743187.f1.docx]

**Online appendix**

1. Correlation between postoperative albumin decrease and intraoperative blood loss


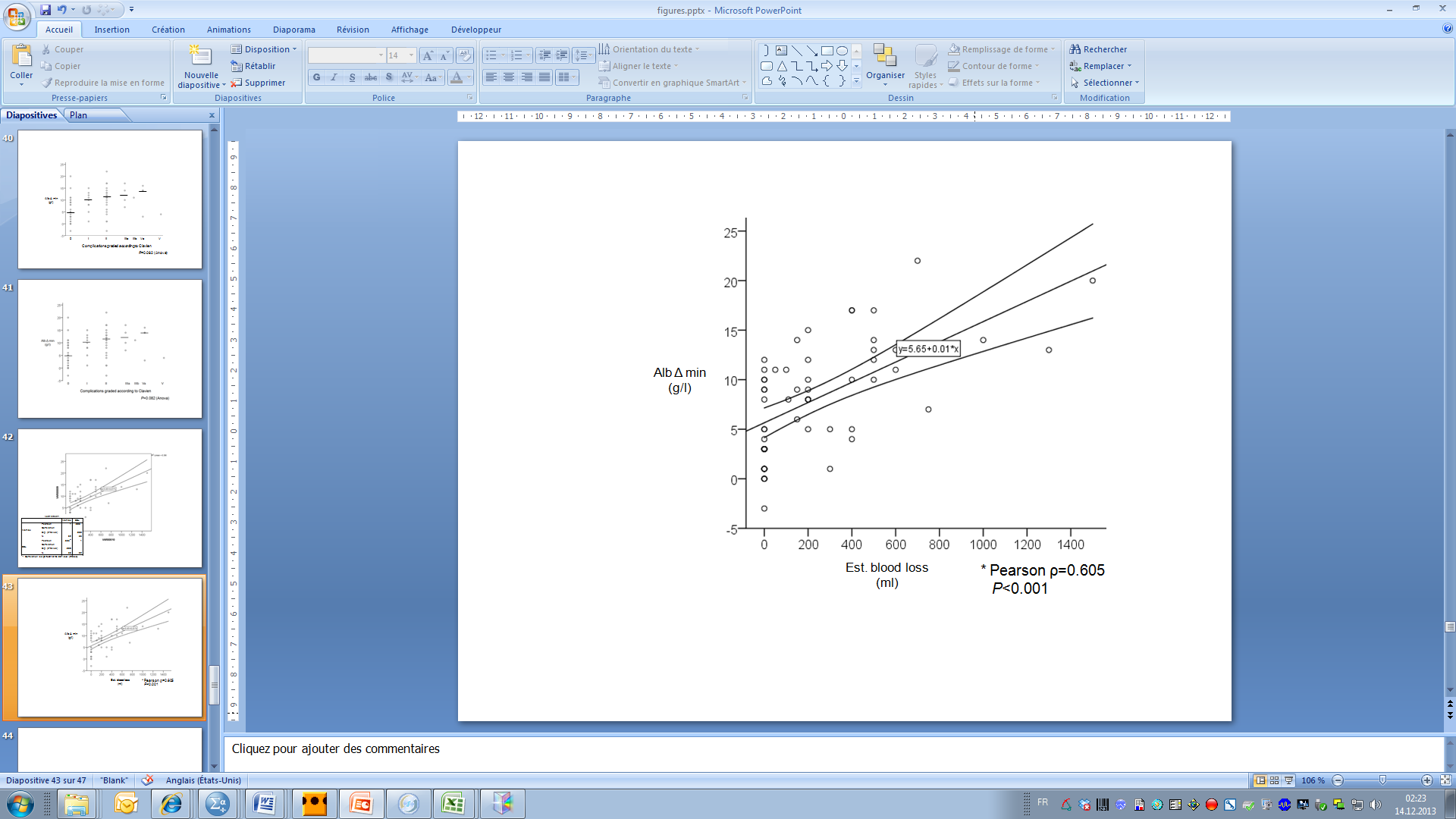


Postoperative albumin decrease (Alb Δ min) is significantly correlated with blood loss.

1. Albumin Δ min values in relation to postoperative complications


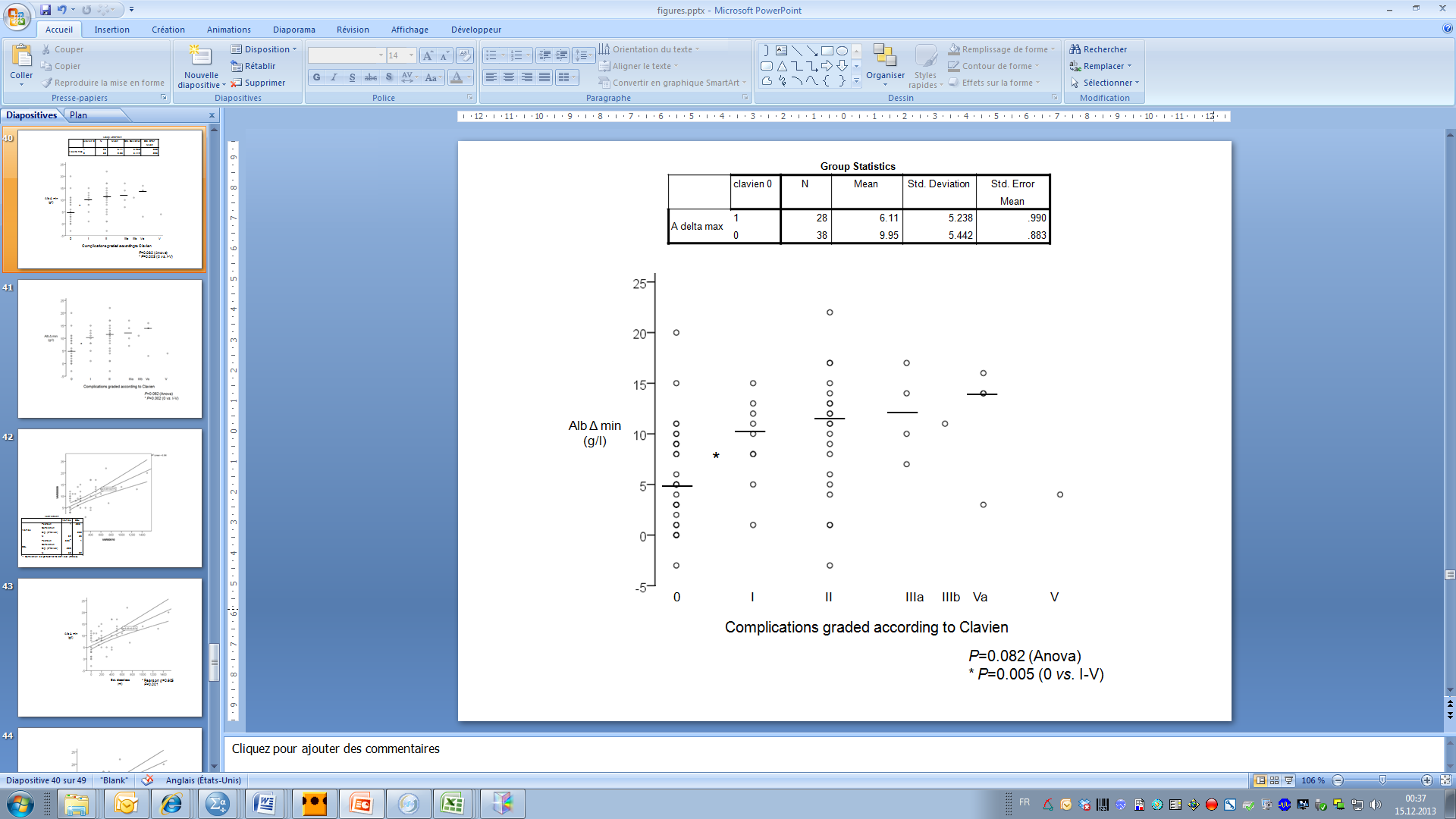


Maximal albumin drop (Alb Δ min) was significantly higher for patients presenting postoperative complications.
